# Nosocomial and Community-Acquired Spontaneous Bacterial Peritonitis in patients with liver cirrhosis in China: Comparative Microbiology and Therapeutic Implications

**DOI:** 10.1038/srep46025

**Published:** 2017-04-06

**Authors:** Lei Shi, Dan Wu, Lei Wei, Suxia Liu, Peng Zhao, Bo Tu, Yangxin Xie, Yanan Liu, Xinhua Wang, Liying Liu, Xin Zhang, Zhe Xu, Fusheng Wang, Enqiang Qin

**Affiliations:** 1 Treatment and Research Center for Infectious Diseases, Beijing 302 Hospital, Beijing, China; 2 Medical Administration Division, Beijing 302 Hospital, Beijing, China; 3 Blood Purification Center, Beijing 302 Hospital, Beijing, China; 4 Department of Laboratory, Beijing 302 Hospital, Beijing, China; 5 Tumor Radiotherapy Center, Beijing 302 Hospital, Beijing, China

## Abstract

Spontaneous bacterial peritonitis (SBP) is a common complication of liver cirrhosis. This study was performed to compare the microbiological characteristics of nosocomial and community-acquired episodes of bacterial peritonitis in China. Five hundred and seventy-five strains were isolated from the ascitic fluid of cirrhotic patients from the Beijing 302 Hospital from January 2014 to December 2014. The patients in the community-acquired SBP (n = 311) and the nosocomial SBP (n = 264) groups exhibited significant differences in clinical symptoms (P < 0.01). In both groups, most of the bacteria were *Escherichia coli*, Klebsiella pneumoniae, coagulase-negative staphylococcus and Enterococcus. There were more frequent gram-positive cocci (G^+^ C) in the nosocomial group (n = 170). Compared with the community-acquired group, the proportion of Enterococcus was significantly increased in the nosocomial group (9.0% vs. 16.6%, P < 0.05). The resistance rate of the main pathogenic bacteria to the recommended first-line drug in the guideline was very high. Community-acquired and nosocomial SBP groups exhibited differences in clinical symptoms and antibiotic susceptibility tests. Optimal treatments should be provided for these patients. We recommend that cefoperazone/sulbactam or piperacillin/tazobactam should be used for the empirical treatment of SBP.

Spontaneous bacterial peritonitis (SBP) is a common complication in cirrhotic patients with ascites^1^,^2^, Patients with ascites who have been followed prospectively for one year have a 10% to 25% incidence of having at least one episode of SBP during that time period^3^. Because of an improved understanding of SBP, an earlier detection of infection, and a larger application of safe and effective antibiotics from which to choose, infection-related mortality resulting from SBP has markedly decreased^4^,^5^. The prognosis is generally improved if antibiotics are started before the onset of shock and renal failure^6^,^7^. However, because of the severe underlying liver disease that is usually a precursor to the development of SBP, the cumulative recurrence rate within the following year is approximately 70% from the first attack of SBP^8^,^9^. Due to the timely diagnosis of infection and following the administration of antibiotics, the mortality rate has decreased to approximately 20–30%^10^. However, the one-year survival rate after recovery from the first episode of SBP is only 30% to 40%^11^.

SBP often occurs during hospitalization and is regarded as a hospital-acquired infection (SBP diagnosis more than 48 hours after hospitalization). Clinically, SBP diagnosed within 48 hours of admission is considered to be community-required SBP. The occurrence of SBP in hospitalized patients is 9% to 17%^11^,^12^ and 1–4%^12^,^13^ in out-patients. In recent years, infections caused by multidrug-resistant bacteria have become an important clinical problem in many countries^14^. Many studies have investigated the treatment efficacy of various antibiotics on SBP and found that the therapeutic consequences were related to the source of SBP infection^10^. Patients with nosocomial SBP exhibited a greater resistance to antibiotics than those with community-acquired SBP^1^,^3^.

Based on the guidelines from American Association for the Study of Liver Diseases(AASLD) and European Association for the Study of the Liver(EASL), cefotaxime or a similar third-generation cephalosporin appears to be the treatment of choice for suspected SBP and is used to cover 95% of the flora, which includes the 3 most common isolates: *Escherichia coli*, Klebsiella pneumoniae, and Streptococcal pneumoniae. Third-generation cephalosporins or quinolones have been suggested as the empirical treatment for SBP^15^,^16^.

*Escherichia coli* of the Enterobacteriaceae family (gram-negative bacilli, G^−^ B) is the main organism that causes SBP^17^. Due to the application of selective decontamination for the prevention of infections by G^−^ B, gram-positive cocci (G^+^ C) are frequently isolated from the ascitic fluid of patients treated by norfloxacin^18^,^19^,^20^. Cheong *et al*. found that the resistance to cephalosporins by G^−^ B was more common in nosocomial SBP than in community-acquired SBP. Moreover, patients who contracted nosocomial SBP had a poorer outcome than patients who contracted community-acquired SBP^21^. This outcome indicated the differences in pathogens between nosocomial and community-acquired SBP and resulted in different treatment strategies.

Based on Chinese population, our study aimed to investigate the bacterial spectrum of nosocomial and community-acquired SBPs and their antibiotic resistance. This outcome contributes to the understanding of the pathogenesis and improved treatment of SBP.

## Materials and Methods

### Subjects

All the infectious agents were isolated from the peritoneal fluid of hospitalized cirrhotic patients in the Beijing 302 Hospital between January 2014 and December 2014. Infectious agents that were isolated from the fluid from peritoneal dialysis or from a secondary bacterial peritonitis were excluded. When the same strain was isolated twice or from more than 1 patient, it was only counted once. This retrospective study analyzed both the types and the drug resistance of bacteria, as well as the total number of polymorphonuclear leukocytes (PMN) in ascitic fluid. The microbiological differences in characteristics between community-acquired and nosocomial SBP were also investigated. This study was approved by the medical ethics council of the Beijing 302 Hospital and written informed consent was obtained from all subjects.

### Diagnostic criteria

SBP was defined as the presence of ≥250 PMN/mm^3^ in ascitic fluid, along with a positive ascitic fluid culture. SBP diagnosis that occurred more than 48 hours after hospitalization was defined as a nosocomial SBP. A diagnosis of SBP within 48 hours of admission without hospitalizations in the preceding 6 months was defined as a community-acquired SBP^15^,^16^.

### Normal tests for ascites

2 ml ascitic fluid samples were extracted under aseptic conditions. The samples were also sent to the laboratory for PMN counting using a modified Neubauer counting chamber under direct microscopy. All methods were conducted in accordance with relevant guidelines and regulations.

### Ascitic culture and antibiotic susceptibility test

10 ml ascitic fluid samples were collected under aseptic conditions and the fluid culture was conducted with both Bact/Alert3D anaerobic and aerobic blood culture bottles (bioMerieux) at the patients’ bedside^22^. The bacteria were inoculated into a Columbia Blood Agar and a China Blue Agar Plate when positive alarm occurred in the culture flask, and then a single colony was isolated and identified using an automated VITEK2 system (bioMerieux). An antibiotic susceptibility test was performed using either the Kirby-Bauer (K-B) or the Minimal Inhibitory Concentration (MIC) method. Quality-control strains included *Escherichia coli* (ATCC25922) and Staphylococcus aureus (ATCC25923). All procedures were conducted in accordance with the guidelines set by the Chinese Society of Laboratory Medicine.

### Statistics

All statistics were analyzed with SPSS version 11.0. The results of antibiotic susceptibility tests were analyzed using WHONET5 software. Continuous variables are expressed as means ± standard deviations (SD). The comparison of continuous variables between groups was conducted using a Student’s t test. χ2 tests were performed to compare the between groups differences in categorical variables. *P* < 0.05 indicated statistical significance.

## Results

### Basic patient information

Enrolled in the study were 575 patients, of which 424 were males and 151 were females. Patients were divided into either nosocomial (n = 264) or community-acquired groups (n = 311) according to the type of infection. Their ages ranged from 15 to 86 years (median: 54 years) and the average age was 53 ± 11.7 years. The causes of cirrhosis included HBV, HCV, alcohol use, PBC, and AIH. There were no significant differences in the causes of cirrhosis between the two groups (P = 0.95). The Child-Pugh Score was also analyzed and the results suggested that there was no difference in scores between groups (P = 0.45). Regarding clinical symptoms, two groups exhibited obvious differences (P < 0.01). Fever, abdominal pain, hepatic encephalopathy, vomiting or diarrhea, and septic shock were more frequently observed in the nosocomial group. Detailed information is shown in Table 1.

### Bacteria distribution in the ascitic fluid of patients

Among the 575 strains of bacteria, there were 293(50.9%) strains of gram-negative bacilli (G^−^ B), 279(48.5%) strains of gram-positive cocci (G^+^ C), and 1 strain of gram-positive bacillus (G^+^ B). In addition, there were also 2 fungal strains. Specific information about the types of bacteria that were found are exhibited in Table 2. In the community-acquired group, the most frequently encountered bacteria, in decreasing order, were as follows: *E. coli* (n = 70, 27.4%), coagulase-negative staphylococci (n = 56, 22%), Klebsiella pneumoniae (n = 35, 13.7%), Enterococcus (n = 23, 9%), and Streptococcus (n = 21, 8.2%). In the nosocomial group, the most frequently encountered bacteria, in decreasing order, were as follows: *E. coli* (n = 83, 25.9%), coagulase-negative staphylococci (n = 75, 23.4%), Klebsiella pneumoniae (n = 40, 12.5%), Enterococcus (n = 53, 16.6%), and Streptococcus (n = 20, 6.2%). There were more gram-positive cocci (G^+^ C) in the nosocomial group (n = 170) than in the community-acquired group (n = 109), P < 0.05. In the statistical analysis, there were no significant differences in the distributions of *Escherichia coli*, Klebsiella pneumoniae, coagulase-negative staphylococcus, and Staphylococcus aureus between the community-acquired and the nosocomial groups. In contrast, significantly different distributions of Enterococci between the two groups were observed. Compared with the community-acquired group, the proportion of Enterococci was significantly increased in the nosocomial group (9.0% vs. 16.6%, P < 0.05).

### Antibiotic susceptibility of G^−^ B bacteria

Antibiotic susceptibility of G^−^ B including *Escherichia coli* and Klebsiella pneumoniae were tested (Table 3, Supplement Table 1). There were obvious differences in the susceptibility of *Escherichia coli* to piperacillin, ceftriaxone and SMZ between the two groups (P < 0.05 for all). In addition, a remarkable difference existed in the susceptibility of Klebsiella pneumoniae to cefepime between the two groups (P < 0.05). In both groups, more than 50% of *Escherichia coli* and Klebsiella pneumoniae were resistant to third-generation cephalosporins or quinolones, but they had a good sensitivity to cefoperazone/sulbactam, piperacillin/tazobactam and carbapenems. In addition, the number of bacteria positive to ESBL was also investigated. Both groups exhibited differences in the number of *Escherichia coli* that were positive to ESBL (P = 0.02). However, obvious differences were not observed with Klebsiella pneumoniae.

### Antibiotic susceptibility of G^+^ C bacteria

Antibiotic susceptibility of G^+^ C bacteria including Staphylococcus aureus, coagulase-negative staphylococcus, and Enterococcus were determined (Table 4, Supplement Table 2). The susceptibility of Staphylococcus aureus to clindamycin in the community-acquired group was stronger than that in the nosocomial group (P < 0.01). For coagulase-negative staphylococcus, its susceptibility to amikacin in the community-acquired group was stronger than that in the nosocomial group (P < 0.05). In contrast, the susceptibility of coagulase-negative staphylococcus to levofloxacin and erythromycin in the nosocomial group was stronger than that in the community-acquired group, (P < 0.05). In both groups, more than 50% of Staphylococcus aureus and coagulase-negative staphylococcus were resistant to third-generation cephalosporins or quinolones, but they had a good sensitivity to Vancomycin, Linezolid and Teicoplanin. Enterococcus also had a good sensitivity to Vancomycin, Linezolid and Teicoplanin.

## Discussion

Antibiotic treatment should be performed immediately after SBP is diagnosed. Therefore, a good understanding of the knowledge of the pathogens involved in these infections is needed to select suitable first-line antibiotics for these organisms. In recent years, an increasing rate of infections with gram-positive cocci (GPC) and multidrug-resistant (MDR) microorganisms has been demonstrated. GPC were the most frequent bacteria in culture-positive cases of SBP and a variety of drug-resistant microorganisms have emerged^23^. Extensively drug-resistant bacteria are an independent predictor of mortality in patients with SBP^24^.

According to the latest guidelines, cefotaxime, or other third-generation cephalosporins or quinolones are the first-line antibiotic for the treatment of patients with SBP. However, Ricart *et al*. reported that amoxicillin-clavulanic acid is an effective alternative to cefotaxime for community-acquired SBP^20^. In their study, only 13.1% of community-acquired pathogens were resistant to amoxicillin-clavulanic acid and no obvious difference with cefotaxime was observed. In contrast, about half of the nosocomial pathogens were resistant to amoxicillin-clavulanic acid. Moreover, nosocomial pathogens were much more resistant to cefotaxime than community-acquired pathogens^21^,^25^. These results suggest that targeted antibiotics should be selected according to the type of SBP infection. In a study conducted by Campillo *et al*., a high proportion of Methicillin-resistant Staphylococcus aureus (MRSA) was observed in nosocomial SBP patients and the combined therapy of vancomycin with a third-generation cephalosporin was administered for empirical treatment^26^.

Beijing 302 hospital is the largest hepatic hospital in China, with patients that come from all over the country. In our study, *Escherichia coli* (G^−^ B) and coagulase-negative staphylococcus (G^+^ C) were the main pathogens in SBP. There were no significant differences in the distribution of these two strains between patients with community-acquired or nosocomial SBP. Klebsiella pneumoniae ranked second in the G^−^ B pathogens in SBP. For G^−^ B pathogens, patients with community-acquired and nosocomial SBP showed differences in the number of strains of Enterobacter sp. Streptococcus was the second most important pathogen in G^+^ C in SBP. For G^+^ C pathogens, patients in the two groups exhibited differences in the distribution of Enterococci. Increases in gram-positive infections and infections with Enterobacteriaceae with antimicrobial resistance have been reported in patients with spontaneous bacterial peritonitis (SBP)^1^,^27^,^28^. Piroth *et al*. identified 229 G^+^ C out of 411 strains from ascitic fluid cultures and found that the main bacteria were coagulase-negative staphylococci (n = 85), Enterococci (n = 54) and Streptococci (n = 50)^29^.

In addition, the antibiotic resistance of G^−^ B and G^+^ C bacteria was investigated. Strains of *Escherichia coli* that were obtained from nosocomial cases were much more resistant to ampicillin than the SBP from community-acquired cases (88.50% vs. 77.10%). In addition, similar results were observed with piperacillin (98.57% vs. 54.22%), ceftriaxone (67.14% vs. 48.19%), and SMZ (87.14% vs. 49.40%). For Klebsiella pneumoniae, its resistance to cefepime in nosocomial cases was much stronger than that in community-acquired cases (20.00% vs. 0.00%). The resistance of Staphylococcus aureus to clindamycin in the community-acquired group was much stronger than that in the nosocomial group. Moreover, coagulase-negative staphylococcus in the community-acquired group showed much more resistance to amikacin than that in the nosocomial group. In contrast, coagulase-negative staphylococcus in the nosocomial group exhibited much more resistance to levofloxacin and erythromycin than that in the community-acquired group. These results suggest that the type of infection in patients with SBP partly determined the antibiotics that were utilized. The resistance rate of the main pathogenic bacteria to the use of the first-line recommended drug in the guideline was very high. This may be related to the unfounded use of antibiotics in China. We recommend that combinations of cefoperazone/sulbactam or piperacillin/tazobactam be used as the empirical treatment for SBP. In nosocomial cases, these antibiotics should be combined with Vancomycin, Linezolid or Teicoplanin, when necessary.

Enrolled in our study were 575 patients who underwent investigation for the effects that the type of SBP infection had on antibiotic resistance. However, the impact that the type of infection had on the patient’s prognosis was not investigated. Future studies should be performed to investigate this issue. Based on our results, further analysis on antibiotic selection should be initiated to confirm the appropriate agents for both community-acquired and nosocomial cases of SBP. In addition, multi-center research may contribute to the procurement of more accurate results, which should be considered in future studies.

In conclusion, community-acquired and nosocomial SBP showed differences in clinical symptoms. *Escherichia coli* and coagulase-negative staphylococcus are dominant pathogens in SBP. G^−^ B pathogens from cases of nosocomial SBP exhibited much more antibiotic resistance than the pathogens from community-acquired cases of SBP. Community-acquired and nosocomial SBP exhibited differences in the distribution of Enterobacter sp. and Enterococcus. Compared with the community-acquired group, the proportion of Enterococcus was significantly increased in the nosocomial group. The resistance rate of the main pathogenic bacteria to the first-line recommended drug in the guideline was very high. Special attention should be paid to the balanced use of antibiotics. We suggest that combinations of cefoperazone/sulbactam or piperacillin/tazobactam are more suitable for the empirical treatment of SBP. In nosocomial cases, these antibiotic combinations should be combined with Vancomycin, Linezolid or Teicoplanin, when necessary. These results may assist in the understanding of the basic characteristics of these two types of SBP and contribute to their appropriate treatment.

## Additional Information

**How to cite this article**: Shi, L. *et al*. Nosocomial and Community-Acquired Spontaneous Bacterial Peritonitis in patients with liver cirrhosis in China: Comparative Microbiology and Therapeutic Implications. *Sci. Rep.* **7**, 46025; doi: 10.1038/srep46025 (2017).

**Publisher's note:** Springer Nature remains neutral with regard to jurisdictional claims in published maps and institutional affiliations.

## Supplementary Material

Supplementary Tables

## Figures and Tables

**Table 1:** Baseline patient data.

	Community-acquired (n = 264)	Nosocomial (n = 311)	P value
Age (in years)	52 ± 11.6	54 ± 11.6	0.41
Male/female	200/64	224/87	0.31
Main cause of cirrhosis (n, %)			0.95
HBV	168 (63.6)	195 (62.7)	
HCV	44 (16.7)	51 (16.4)	
Alcohol use	34 (12.9)	40 (12.9)	
PBC	11 (4.2)	18 (5.8)	
AIH	4 (1.5)	5 (1.6)	
Unknown	3 (1.1)	2 (0.6)	
Child-Pugh Score (n, %)			0.45
A	0	0	
B	64 (24.2)	84 (27)	
C	200 (75.8)	227 (73)	
Clinical findings (n, %)			<0.01
Fever	202 (76.5)	262 (84.2)	
Abdominal pain	143 (54.1)	201 (64.6)	
Hepatic encephalopathy	92 (34.8)	214 (68.8)	
Vomiting/diarrhea	62 (23.4)	118 (37.9)	
Septic shock	39 (14.8)	65 (20.9)	

**Table 2:** Bacteria distributions in ascitic fluid.

	Total (n, %)	Community-acquired (n, %)	Nosocomial (n, %)	P value
G^−^ B	293	148	145	0.92
*Escherichia coli*	153 (26.6)	70 (27.4)	83 (25.9)	0.68
*Klebsiella pneumoniae*	75 (13.0)	35 (13.7)	40 (12.5)	0.66
*Acinetobacter baumannii*	7 (1.2)	5 (2.0)	2 (0.6)	0.15
*Pseudomonas aeruginosa*	4 (0.7)	2 (0.8)	2 (0.6)	0.82
*Enterobacter sp.*	17 (3.0)	12 (4.7)	5 (1.6)	0.03
*Serratia marcescens*	2 (0.3)	1 (0.4)	1 (0.3)	0.87
*Serratia fonticola*	2 (0.3)	2 (0.8)	0 (0.0)	0.11
*Stenotrophomonas maltophilia*	3 (0.5)	0 (0.0)	3 (0.9)	0.12
*Pseudomonas putida*	3 (0.5)	3 (1.2)	0 (0.0)	0.05
*Bacterium vulgare*	3 (0.5)	2 (0.8)	1 (0.3)	0.44
*Proteus mirabilis*	1 (0.2)	0 (0.0)	1 (0.3)	0.37
*Burkholderia cepacia*	3 (0.5)	2 (0.8)	1 (0.3)	0.44
*Acinetobacter lwoffii*	1 (0.2)	1 (0.4)	0 (0.0)	0.26
*Salmonella*	4 (0.7)	3 (1.2)	1 (0.3)	0.22
*Shigella spp*	5 (0.9)	4 (1.6)	1 (0.3)	0.11
*Morganella morganii*	3 (0.5)	1 (0.4)	2 (0.6)	0.70
*Citrobacter*	3 (0.5)	1 (0.4)	2 (0.6)	0.70
*Haemophilus influenzae*	2 (0.3)	2 (0.8)	0 (0.0)	0.11
*Achromobacter xylosoxidans*	2 (0.3)	2 (0.8)	0 (0.0)	0.11
G^+^ C	279	109	170	0.04
*Coagulase-negative staphylococcus*	131 (22.8)	56 (22.0)	75 (23.4)	0.68
*Staphylococcus aureus*	30 (5.2)	9 (3.5)	21 (6.6)	0.10
*Staphylococcus lugdunensis*	1 (0.2)	0 (0.0)	1 (0.3)	0.37
*Faecalis*	52 (9.0)	15 (5.8)	−37 (11.7)	<0.01
*Faecium*	18 (1.7)	5 (1.9)	13 (4.1)	<0.05
Other *Enterococcus*	6 (1.0)	1 (0.3)	5 (1.6)	<0.05
*Streptococcus Pneumoniae*	1 (0.2)	1 (0.4)	0 (0.0)	0.26
*Streptococcus* (other than *S. pneumoniae*)	40 (7.0)	20 (7.8)	20 (6.2)	0.46
G^+^ B	1	1	0	
*Ochrobactrum Anthropic*	1 (0.2)	1 (0.4)	0 (0.0)	0.26
*Anaerobic bacteria*	0 (0.0)	0 (0.0)	0 (0.0)	—
*Fungus*	2	0	2	
*Candida glabrata*	2 (0.3)	0 (0.0)	2 (0.6)	0.21

**Table 3:** Antibiotic resistance of G^−^ B bacteria (strains, n).

Antibiotics	*Escherichia coli*				P	*Klebsiella pneumoniae*				P
	Community-acquired (n = 83)		Nosocomial (n = 70)			Community-acquired (n = 40)		Nosocomial (n = 35)		
	ESBL positive (n = 39)	ESBL negative (n = 44)	ESBL positive (n = 46)	ESBL negative (n = 24)		ESBL positive (n = 15)	ESBL negative (n = 25)	ESBL positive (n = 10)	ESBL negative (n = 25)	
Ampicillin	35	29	46	16	0.06	11	24	10	23	0.31
Piperacillin	28	17	37	12	<0.01	11	13	10	6	0.22
Ticarcillin/clavulanic acid	37	15	44	9	0.08	9	10	7	12	0.56
Piperacillin/tazobactam	12	6	8	1	0.15	1	1	4	0	0.31
Cefoperazone	38	3	37	3	0.34	5	1	10	1	0.09
Cefoperazone/Sulbactam	10	1	11	2	0.37	2	0	3	1	0.31
Ceftazidime	32	2	30	1	0.68	9	0	10	0	0.55
Ceftriaxone	38	2	45	2	0.02	11	0	10	0	0.92
Cefepime	26	2	29	3	0.33	0	0	6	1	<0.01
Cefmetazole	8	4	4	6	0.98	9	0	2	3	0.36
Aztreonam	35	2	33	1	0.62	11	0	8	1	0.86
Imipenem	1	0	2	0	0.46	0	0	1	0	0.28
Meropenem	1	0	1	0	0.90	0	0	1	0	0.28
Amikacin	5	1	4	1	0.98	1	1	3	1	0.31
Levofloxacin	30	10	31	10	0.20	9	1	4	4	0.83
SMZ	29	12	35	22	<0.01	9	2	7	4	0.71
Fosfomycin	11	2	3	3	0.18	2	7	3	2	0.36

**Table 4:** Antibiotic resistance of G^+^ C bacteria (strains, n).

Antibiotics	Staphylococcus aureus (n = 30)				P	Coagulase negative staphylococcus (n = 131)				P	Enterococcus (n = 76)	
	Community-acquired (n = 9)		Nosocomial (n = 21)			Community-acquired (n = 56)		Nosocomial (n = 75)			Nosocomial (n = 76)	
	MRSA (n = 6)	MSSA (n = 3)	MRSA(n = 6)	MSSA (n = 15)		MRSCON (n = 40)	MSSCON (n = 16)	MRSCON (n = 61)	MSSCON (n = 14)		*Faecalis* (n = 52)	*Faecium* (n = 18)
SBL	6	3	6	15	—	40	9	61	7	0.56	22	5
Penicillin G	6	3	6	15	—	40	9	61	7	0.56	48	5
Ceftriaxone	6	0	6	0	0.05	40	0	61	0	0.18	—	
Cefoxitin	6	0	6	0	0.05	40	0	61	0	0.18	—	
Amikacin	3	0	4	2	0.79	17	0	5	0	<0.01	—	
Levofloxacin	4	2	6	2	0.15	11	2	34	0	<0.01	39	5
SMZ	2	0	2	1	0.59	20	6	35	1	0.86	—	
Clindamycin	4	3	2	5	<0.01	18	5	36	1	0.35	—	
Erythromycin	3	2	2	10	0.94	25	11	54	7	0.03	41	11
Vancomycin	0	0	0	1	0.51	0	0	0	0	—	0	0
Linezolid	0	0	0	0	—	1	0	0	0	0.24	1	3
Teicoplanin	0	0	0	0	—	0	0	2	0	0.22	1	0
Tetracycline	3	0	4	2	0.79	10	4	15	3	0.90	48	11
