# Corrigendum: Nosocomial and Community-Acquired Spontaneous Bacterial Peritonitis in patients with liver cirrhosis in China: Comparative Microbiology and Therapeutic Implications

**DOI:** 10.1038/srep46868

**Published:** 2017-07-27

**Authors:** Lei Shi, Dan Wu, Lei Wei, Suxia Liu, Peng Zhao, Bo Tu, Yangxin Xie, Yanan Liu, Xinhua Wang, Liying Liu, Xin Zhang, Zhe Xu, Fusheng Wang, Enqiang Qin

Scientific Reports 7: Article number: 46025; 10.1038/srep46025 published online: 04 06 2017; updated: 07 27 2017.

The original version of this Article contained errors. In the Abstract,

“The patients in the community-acquired SBP (n = 311) and the nosocomial SBP (n = 264) groups exhibited significant differences in clinical symptoms (P < 0.01)”.

now reads:

“The patients in the community-acquired SBP (n = 264) and the nosocomial SBP (n = 311) groups exhibited significant differences in clinical symptoms (P < 0.01)”.

In the Results section, under subheading ‘Basic patient information’,

“Patients were divided into either nosocomial (n = 264) or community-acquired groups (n = 311) according to the type of infection”.

now reads:

“Patients were divided into either nosocomial (n = 311) or community-acquired groups (n = 264) according to the type of infection”.

As a result, the values of the columns ‘Community-acquired (n, %)’ and ‘Nosocomial (n, %)’ in Table 2 were incorrect.

These errors have now been corrected in the PDF and HTML versions of the Article.

## Figures and Tables

**Table 2:** Bacteria distributions in ascitic fluid.

	**Total (n, %)**	**Community-acquired (n, %)**	**Nosocomial (n, %)**	**P value**
**G**−**B**	**293**	**156**	**137**	**0.92**
*Escherichia coli*	153 (26.6)	78 (50)	75 (54.7)	0.68
*Klebsiella pneumoniae*	75 (13.0)	35 (22.4)	40 (29.2)	0.66
*Acinetobacter baumannii*	7 (1.2)	5 (3.2)	2 (1.6)	0.15
*Pseudomonas aeruginosa*	4 (0.7)	2 (1.3)	2 (1.6)	0.82
*Enterobacter sp.*	17 (3.0)	12 (7.7)	5 (3.6)	0.03
*Serratia marcescens*	2 (0.3)	1 (0.6)	1 (0.7)	0.87
*Serratia fonticola*	2 (0.3)	2 (1.3)	0 (0.0)	0.11
*Stenotrophomonas maltophilia*	3 (0.5)	0 (0.0)	3 (2.2)	0.12
*Pseudomonas putida*	3 (0.5)	3 (1.9)	0 (0.0)	0.05
*Bacterium vulgare*	3 (0.5)	2 (1.3)	1 (0.7)	0.44
*Proteus mirabilis*	1 (0.2)	0 (0.0)	1 (0.7)	0.37
*Burkholderia cepacia*	3 (0.5)	2 (1.3)	1 (0.7)	0.44
*Acinetobacter lwoffii*	1 (0.2)	1(0.6)	0 (0.0)	0.26
*Salmonella*	4 (0.7)	3 (1.9)	1 (0.7)	0.22
*Shigella spp*	5 (0.9)	4 (2.6)	1 (0.7)	0.11
*Morganella morganii*	3 (0.5)	1 (0.6)	2 (1.5)	0.70
*Citrobacter*	3 (0.5)	1 (0.6)	2 (1.5)	0.70
*Haemophilus influenzae*	2 (0.3)	2 (1.3)	0 (0.0)	0.11
*Achromobacter xylosoxidans*	2 (0.3)	2 (1.3)	0 (0.0)	0.11
**G**+**C**	**279**	**107**	**172**	**0.04**
*Coagulase-negative staphylococcus*	131 (22.8)	56 (52.3)	75 (43.6)	0.68
*Staphylococcus aureus*	30 (5.2)	9 (8.4)	21 (12.2)	0.10
*Staphylococcus lugdunensis*	1 (0.2)	0 (0.0)	1 (0.6)	0.37
*Faecalis*	52 (9.0)	15 (14.0)	37 (21.5)	<0.01
*Faecium*	18 (1.7)	5 (4.7)	13 (7.6)	<0.05
Other *Enterococcus*	6 (1.0)	1 (0.9)	5 (2.9)	<0.05
*Streptococcus Pneumoniae*	1 (0.2)	1 (0.9)	0 (0.0)	0.26
*Streptococcus (*other than *S. pneumoniae)*	40 (7.0)	20 (18.7)	20 (11.6)	0.46
**G**+**B**	**1**	**1**	**0**	
*Ochrobactrum Anthropic*	1 (0.17)	1	0	0.26
*Anaerobic bacteria*	0 (0.0)	0	0	—
***Fungus***	**2**	**0**	**2**	
*Candida glabrata*	2 (0.3)	0	2	0.21

