# Supplementary material for: Nosocomial and Community-Acquired Spontaneous Bacterial Peritonitis in patients with liver cirrhosis in China: Comparative Microbiology and Therapeutic Implications
Source: Sci Rep. 2017 Apr 6;7:46025. doi: 10.1038/srep46025 (PMC5382543; doi:10.1038/srep46025)
Supplement: Supplementary Tables [file srep46025-s1.pdf]

Nosocomial and Community-Acquired Spontaneous Bacterial Peritonitis in patients with liver cirrhosis in China: Comparative Microbiology and Therapeutic Implications

Lei Shi[1*], Dan Wu[1*], Lei Wei[2], Suxia Liu[3], Peng Zhao[1], Bo Tu[1], Yangxin Xie[1], Yanan Liu[4], Xinhua Wang[1], Liying Liu[5], Xin Zhang[1], Zhe Xu[1], Fusheng Wang[1#], Enqiang Qin[1#]

1. Treatment and Research Center for Infectious Diseases, Beijing 302 Hospital, Beijing, China.
2. Medical Administration Division, Beijing 302 Hospital, Beijing, China.
3. Blood Purification Center, Beijing 302 Hospital, Beijing, China.
4. Department of Laboratory, Beijing 302 Hospital, Beijing, China.
5. Tumor Radiotherapy Center, Beijing 302 Hospital, Beijing, China.

#Correspondence:

Enqiang Qin, Ph.D., M.D., Treatment and Research Center for Infectious Diseases, 100 the Western 4th Ring Middle Road, Beijing 302 Hospital, Beijing 100039, China.

Email: qeq2004@sina.com

FuSheng Wang, Ph.D., M.D., Treatment and Research Center for Infectious Diseases, 100 the Western 4th Ring Middle Road, Beijing 302 Hospital, Beijing 100039, China.

Email: fswang302@163.com

* The authors equally contributed to this work.

Supplement Table 1. Antibiotic sensitive of G- B bacteria (strains, n)

Antibiotics	*Escherichia coli*				*Klebsiella pneumoniae*			
	Community-acquired (n=83)		Nosocomoal (n=70)		Community-acquired (n=40)		Nosocomoal (n=35)	
	ESBL positive (n=39)	ESBL negative (n=44)	ESBL positive (n=46)	ESBL negative (n=24)	ESBL positive (n=15)	ESBL negative (n=25)	ESBL positive (n=10)	ESBL negative (n=25)
Ampicillin	3	15	0	8	4	1	0	2
Piperacillin	9	26	8	12	4	12	0	19
Ticarcillin/clavulanic acid	1	29	2	15	6	13	3	13
Piperacillin/tazobactam	26	37	38	23	13	24	6	25
Cefoperazone	1	40	9	21	10	24	0	23
Cefoperazone/Sulbactam	29	43	35	22	3	25	7	23
Ceftazidime	6	41	15	22	6	25	0	25
Ceftriaxone	0	42	1	21	4	25	0	25
Cefepime	12	42	17	21	15	25	4	24
Cefmetazole	31	40	42	18	6	25	7	22
Aztreonam	4	42	12	23	4	25	1	24
Imipenem	38	43	44	24	15	25	9	25
Meropenem	38	44	45	24	15	25	9	25
Amikacin	34	43	41	23	14	24	7	24
Levofloxacin	9	33	15	13	6	23	6	20
SMZ	9	31	10	2	6	23	3	21
Fosfomycin	27	42	43	21	12	18	7	23

Supplement Table 2. Antibiotic Sensitive of G+ C bacteria (strains, n)

Antibiotics	Staphylococcus aureus（n=30）				Coagulase negative staphylococcus（n=131）				Enterococcus (n=76)	
	Community-acquired (n=9)		Nosocomoal (n=21)		Community-acquired (n=56)		Nosocomoal (n=75)		Nosocomoal (n=76)	
	MRSA (n=6)	MSSA (n=3)	MRSA (n=6)	MSSA (n=15)	MRSCON (n=40)	MSSCON (n=16)	MRSCON (n=61)	MSSCON (n=14)	*Faecalis* (n=52)	*Faecium* (n=18)
SBL	0	0	0	0	0	7	0	7	22	13
Penicillin G	0	0	0	0	0	7	0	7	48	12
Ceftriaxone	0	3	0	15	0	15	0	14	-	-
Cefoxitin	0	3	0	15	0	16	0	13	-	-
Amikacin	3	3	2	13	22	16	56	12	-	-
Levofloxacin	2	1	0	13	29	14	27	13	39	13
SMZ	3	3	4	14	20	10	25	12	-	
Clindamycin	2	0	3	10	22	11	25	13	-	
Erythromycin	3	1	4	5	15	5	6	7	8	7
Vancomycin	6	3	6	14	40	16	61	14	52	17
Linezolid	6	3	6	15	39	16	60	14	51	15
Teicoplanin	6	3	6	15	40	16	59	14	51	18
Tetracycline	3	3	2	13	30	12	45	11	2	7
